# The burden of liver cancer in Mongolia from 1990–2019: a systematic analysis for the Global Burden of Disease Study 2019

**DOI:** 10.3389/fonc.2024.1381173

**Published:** 2024-09-03

**Authors:** Oyundari Batsaikhan, Odgerel Chimed-Ochir, Tatsuhiko Kubo, Chinburen Jigjidsuren, Vanya Delgermaa, Anuzaya Purevdagva, Amarzaya Sarankhuu, Erdenekhuu Nansalmaa, Uranchimeg Tsegmed, Badral Davgasuren, Oyuntsetseg Purev, Ali H. Mokdad, Nicole Davis Weaver, Ryenchindorj Erkhembayar, Christopher J. L. Murray, Mohsen Naghavi

**Affiliations:** ^1^ Department of Public Health and Health Policy, Hiroshima University, Hiroshima, Japan; ^2^ State Great Hural, Parliament of Mongolia, Ulaanbaatar, Mongolia; ^3^ Independent Researcher, Ulaanbaatar, Mongolia; ^4^ Communicable Diseases, World Health Organization (WHO), Ulaanbaatar, Mongolia; ^5^ Mongolian Field Epidemiologists Society, Ulaanbaatar, Mongolia; ^6^ National Cancer Center of Mongolia, Ulaanbaatar, Mongolia; ^7^ Infectious Diseases Surveillance and Research Department, National Center for Communicable Diseases, Ulaanbaatar, Mongolia; ^8^ Department of Policy Planning, Ministry of Health, Ulaanbaatar, Mongolia; ^9^ Institute for Health Metrics and Evaluation, University of Washington, Seattle, WA, United States; ^10^ Department of Health Metrics Sciences, School of Medicine, University of Washington, Seattle, WA, United States; ^11^ Department of International Cyber Education, Mongolian National University of Medical Sciences, Ulaanbaatar, Mongolia

**Keywords:** liver cancer (LC), viral hepatitis, hepatitis B, hepatitis C, alcohol, GBD

## Abstract

**Background:**

Liver cancer remains the leading cause of death and public health threat among the Mongolian population. So far, there has been no in-depth analysis to describe the burden of common attributable factors to liver cancer in Mongolia. Therefore, we aimed to explore the most prevalent causes of liver cancer and its trends from 1990 to 2019.

**Methods:**

We extracted the primary liver cancer data from the Global Burden of Diseases, Injuries, and Risk Factors Study (GBD) 2019 to examine the mortality and morbidity of liver cancer by its etiological types, which included alcohol, viral hepatitis B and C, and non-alcoholic steatohepatitis (NASH). The data was extracted by sex and 5-year age intervals from 1990 to 2019. Data included mortality, incidence, years of life lost (YLLs), years lived with disability (YLDs), and disability-adjusted life-years (DALYs) of liver cancer among the Mongolian population.

**Results:**

Mongolia had the world’s highest age-standardized DALYs for liver cancer (2558.1) in 2019. Alcohol-attributable DALYs (786.6) were 29 times higher than the global average (26.1), and liver cancer due to hepatitis C (752.6) and B (763.2) were 21.5 (35.0) and 10.9 (69.1) times higher, respectively. Over the past 30 years, there has been a steady increase in the incidence and number of deaths caused by liver cancer in Mongolia. In 2019, liver cancer incidence due to alcohol consumption was 3.1 times higher for males than females, and hepatitis B was 2.7 times higher for males than females. However, the incidence of hepatitis C and NASH were slightly higher for females. Deaths from liver cancer accounted for 9.51% (2365) of total deaths in Mongolia in 2019, with a continuously increasing trend in the fraction of death compared to 1990, which was 11 times higher than the global average (0.86%), particularly in females with a 319.6% (95% UI 234.9–435.7) increase observed during the study period. Liver cancer due to hepatitis B, C, and alcohol each shared about one-third of liver cancer deaths.

**Conclusion:**

A comprehensive analysis of the burden of liver cancer in Mongolia reveals alcohol use as a primary cause of liver cancer mortality, particularly affecting men and significantly impacting the disease burden. Viral hepatitis continues to pose a major public health concern in the country. Although significant milestones have progressed, addressing the unique demographic and geographical challenges requires tailored approaches for specific target populations. The evidence generated from this analysis is crucial to support policy guidance, contribute to evidence-based decisions, guide public health prevention measures, and amplify population health promotion and disease prevention throughout Mongolia.

## Introduction

Mongolia is a landlocked country in Eastern Asia with a total population of over 3.2 million. It ranks as one of the least densely populated countries, with a vast terrain and harsh climate and geographical conditions. More than half of the population resides in urban areas, and only 8.6% are herders, living a nomadic lifestyle in the countryside (1). The current health system has two tiers, primary and referral care levels based on the centralized Semashko system, which was modified since the democratic revolution in 1990. The health reform introduced universal health coverage at all levels of health facilities to offer quality care services to the population overcoming financial and geographical barriers (2). As a result of social, economic, and health systems development, the average life expectancy has increased substantially in the past decade, along with shifts in disease burden (3, 4).

Remarkable achievements around maternal-and-child health, under-5 mortality, and many communicable and non-communicable diseases are sustained through political commitments and the successful implementation of various national programs. Despite these efforts, liver cancer, cirrhosis, and other chronic liver diseases remained as leading causes of death with a continuous increase over the past decades (4, 5). Numerous studies have been conducted to determine the prevalence of the epidemic and its causes in Mongolia (6–9). According to the analysis of Mongolia’s health situation based on GBD 2019, liver cancer death had the highest increase in the age-standardized rate, which was 20 times higher than the global rates. Specifically, liver cancer death due to alcohol use was 31 times higher than the global rate, followed by hepatitis B, which was 23 times higher and hepatitis C, which was 12 times higher (4).

In 2011, Baatarkhuu et al. reported that Mongolia was having liver cancer patients diagnosed at an advanced stage, contributing to lower survival rates which are observed until today (6). More than half of the patients with acute or chronic hepatitis were either HBsAg or anti-HCV positive (8). The main risk factors for the high prevalence of viral hepatitis and its attribution to liver cancer were explained by insufficient infection control practices such as dental treatment, glass syringe use at home, surgery, and blood transfusion (7). Adding to the current evidence, we aim to analyze the burden of the most common attributable factors to liver cancer, including viral hepatitis, alcohol, and non-alcoholic steatohepatitis, and estimated disability-adjusted life-years (DALYs) for the first time among the Mongolian population. This study can be used to guide policy, contribute to evidence-based decisions and public health prevention measures, and amplify population health promotion and disease prevention throughout Mongolia.

## Methods

We extracted the primary liver cancer data and subgroups of liver cancer including liver cancers due to hepatitis B, hepatitis C, alcohol, NASH, and other causes from the Global Burden of Diseases, Injuries, and Risk Factors Study (GBD) 2019 (10). The data was extracted by sex and 5-year age intervals from 1990 to 2019. Data included mortality, incidence, prevalence, years lived with disability (YLDs), years of life lost (YLLs), and disability-adjusted life-years (DALYs) of liver cancer (11). Cancer mortality data including vital registration, verbal autopsy, and cancer registry data were used in the analysis. Before the integration of the cancer registry data into the cause of death database of GBD, it went through 12 processing steps (11). Liver cancer mortality modeling followed the Cause of Death Ensemble model (CODEm) process. Then, liver cancer was grouped into five etiology groups, including liver cancer due to hepatitis B, liver cancer due to hepatitis C, liver cancer due to alcohol, liver cancer due to non-alcoholic steatohepatitis, and liver cancer due to other causes. To find the proportion of liver cancer cases due to these five etiology groups, a systematic literature search was performed in PubMed on 24 October 2016. For each relevant study, the proportions of liver cancer due to the five specific risk factors were calculated and were estimated with adjusting respective covariates including alcohol consumption, HIV age-standardized prevalence, the seroprevalence of HBsAg and anti-HCV (Level 1), the population coverage of three-dose hepatitis B vaccination, intravenous drug use, cumulative cigarettes, mean BMI, healthcare access and quality index, diabetes fasting plasma glucose (Level 2), education, age-and sex-specific summary exposure value for high red meat, LDI, and socio-demographic index (Level 3). After adjusting these data with the aforementioned covariates using the CODEm, the GBD estimates were obtained using a Bayesian meta-regression modeling tool, DisMod-MR 2.1 for both sexes, years from 1990 to 2019, and all age groups. Details on the estimation of the proportion of liver cancer cases due to these five etiology groups have been described elsewhere (11).

DALYs for a specific cause are calculated as the sum of YLLs due to premature mortality from that cause and YLDs due to disability for people living in states of less than good health resulting from the specific cause. YLLs are calculated by subtracting the age at death from the longest possible life expectancy for a person at that age of given country. It is measured by taking the prevalence of the condition multiplied by the disability weight for that condition (12). The weights are measured on a scale from 0 to 1, where 0 equals a state of full health and 1 equals death. GBD provided disability weights for the 440 health states (including combined health states) to estimate nonfatal health outcomes for the GBD 2019 study (13, 14). ICD-10 codes C22–22.8 and D13.4 were used for primary liver cancer.

We reported all rates as age-standardized rates derived from world population standards that were developed for the GBD study, and each point estimate of percentage change in age adjusted mortality rate includes 95% uncertainty intervals (UIs). The trends of liver cancer incidence were analyzed by its five etiological types and reported for males and females. Trends in age-standardized mortality rates and percentage changes from 1990 to2010 and 2010–2019 are provided for liver cancer and its five etiological types for males and females. We also show the age distribution of liver cancer death by each subtype. DALYs and the percentage change of age-standardized rates of DALYs were also compiled for each subtype of liver cancer.

## Results

**Figure 1:**
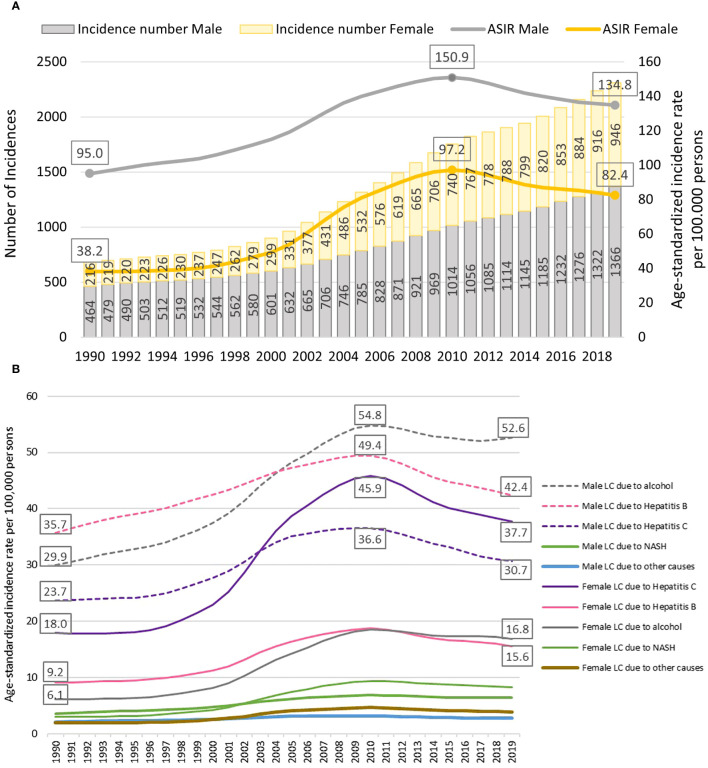
**(A)** Number of incidences and age-standardized incidence rate of liver cancer by sex, 1990–2019. **(B)** Age-standardized incidence rate of liver cancer by its etiologies, 1990–2019. LC: liver cancer; NASH: nonalcoholic steatohepatitis.

**Figure 1A** shows the number of incidences and age-standardized incidence rate of liver cancer for the years 1990–2019. The number of incidences due to liver cancer had a steady increase in the past 30 years. As of 2019, a total of 2312 new cases of liver cancer (1366 for males and 946 for females) occurred in Mongolia. The age-standardized incidence rate also gradually increased for both males and females between 1990 and 2010, but declined slightly between 2010–2019 for both sexes. The total number of incidences (ranging from 464–1,366 among men and 216–946 among women from 1990 to 2019) and age-standardized incidence rate of liver cancer (ranging from 95.0–134.8 among men and 38.2–82.4 among women from 1990 to 2019) for males is 1.4–2.6 times higher than for females throughout the entire study period.

**Figure 1B** shows the age-standardized incidence rate of liver cancer by its etiologies. The incidence rate of liver cancer due to alcohol and hepatitis B was consistently higher in males than in females throughout the entire study period. In 2019, liver cancer due to alcohol was 3.1 times higher, and hepatitis B was 2.7 times higher for males than females. Liver cancer due to hepatitis C was higher in males until 2002 when the rate for females began to surpass the rate for males. Similarly, liver cancer due to nonalcoholic steatohepatitis (NASH) was higher in males until 2001, and afterward, the incidence rate was higher in females. **Table 1** shows the number of deaths from liver cancer from1990 to 2019 in Mongolia. As of 2019, a total of 2365 deaths from liver cancer occurred which accounted for 9.5% of total all- cause deaths in Mongolia with a continuous increasing trend in the fraction of liver cancer deaths from 1990 to 2019 (percent change [PC]=183.5% for male and PC=319.6% for female) (**Supplementary Figure 1**). **Table 1** also depicts that overall, liver cancer due to hepatitis B, C, and alcohol were each responsible for about one-third of liver cancer deaths. The majority of liver cancers in males in 2019 were attributable to alcohol use (38.1%) and hepatitis B (36.1%), whereas liver cancer due to hepatitis C accounted for a larger proportion (43.4%) of liver cancer for females. All types of liver cancer substantially increased from 1990 to 2010, particularly for females, and then started to decline except for the alcohol-attributed liver cancer incidence, although it is not significant.

**Table 1:** Number and age-standardized mortality rate of liver cancer deaths in 2019 and etiologies and their percentage changes from 1990 to 2019 in Mongolia.

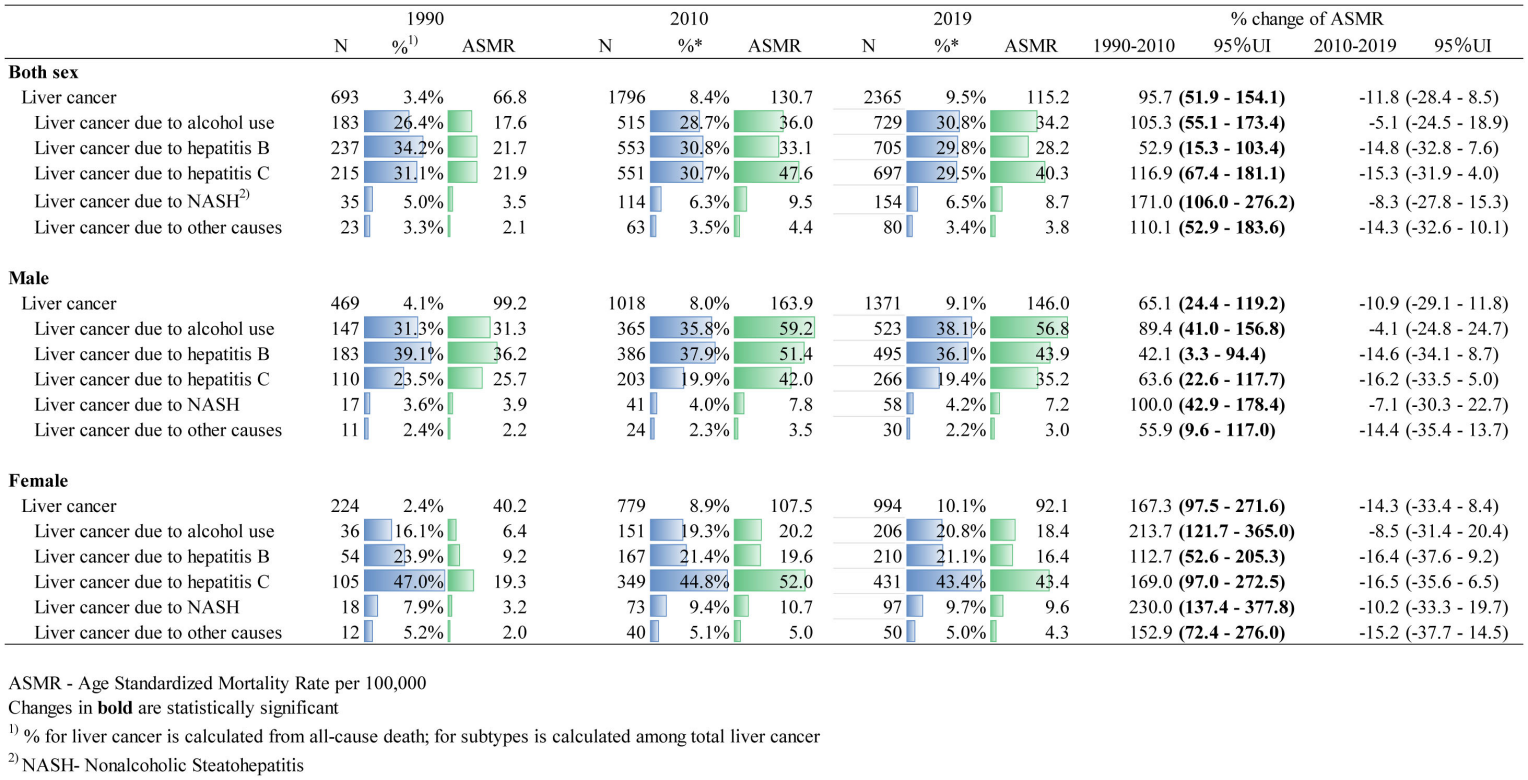	

**Figure 2:**
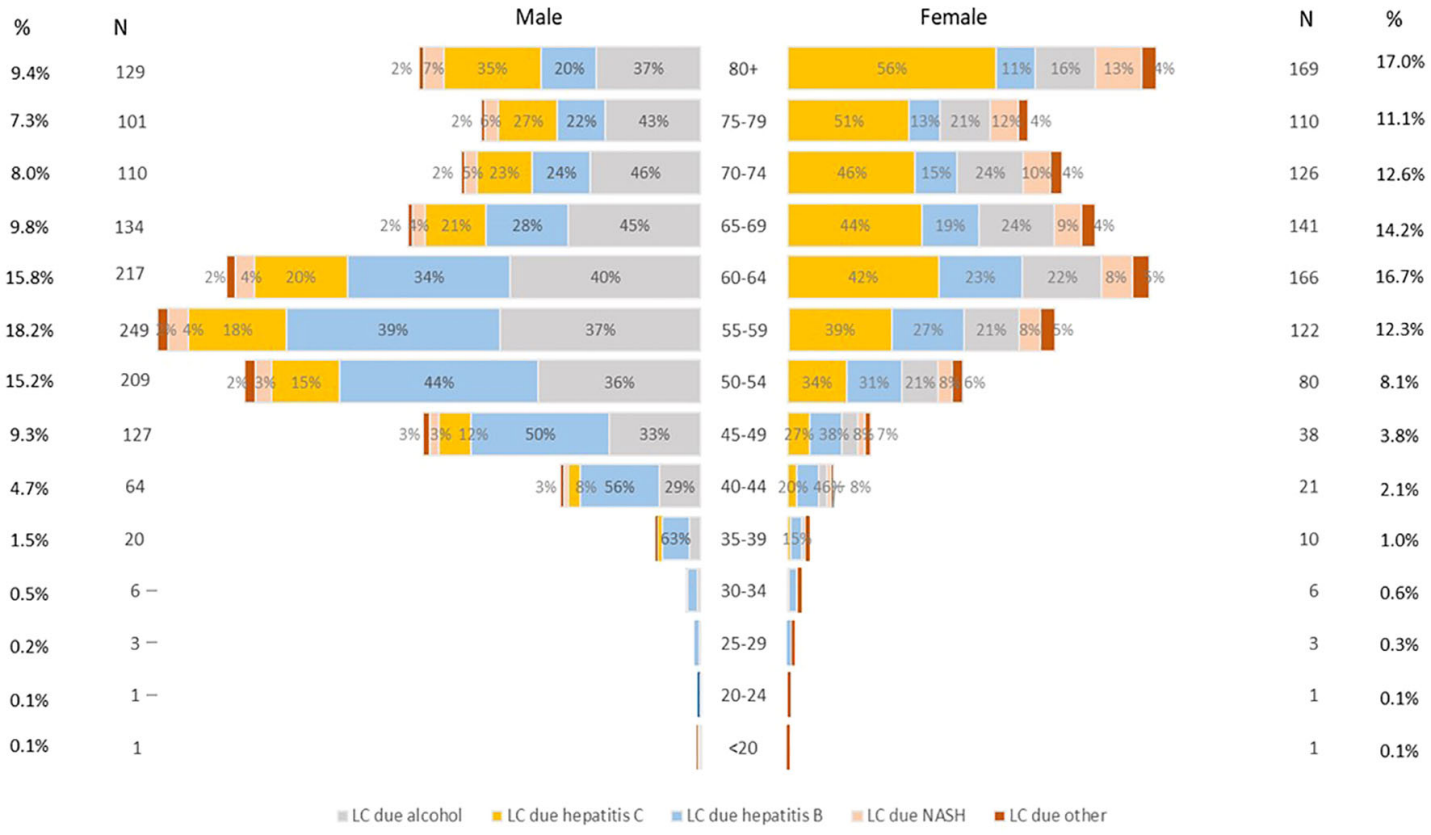
Comparison of age distribution of liver cancer death in males and females in 2019.

**Figure 2** shows the age distribution of liver cancer deaths in 2019. More than half (59%) of liver cancer deaths in males occurred between the ages of 50–69, while more than two-thirds (72%) in females occurred over the age of 60. However, males and females over 70 years old had the highest death rate for all etiologies (**Supplementary Figure 2**). Deaths from liver cancer due to hepatitis B and alcohol use were higher for males than for females for all age groups, whereas deaths from liver cancer due to hepatitis C were higher for females than males. Similarly, liver cancer deaths due to NASH and other causes were higher among females. For the 34 and younger age group, there were relatively fewer cases with a higher percentage of hepatitis B observed.

**Figure 3:**
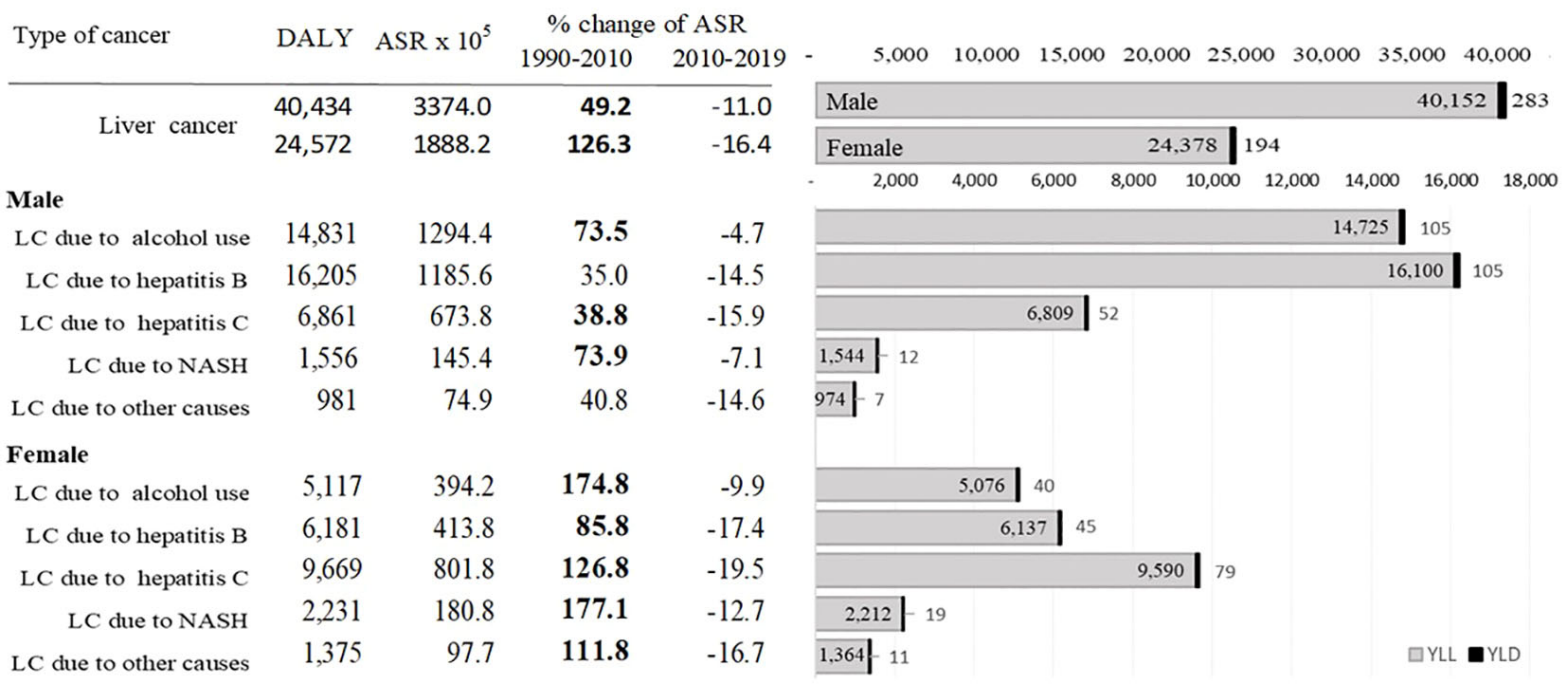
DALYs due to liver cancer from 1990 to 2019 in Mongolia. ASR: age-standardized rate; DALYs: disability-adjusted life years; YLLs: years of life lost.

**Figure 3** shows DALYs due to liver cancer from 1990 to 2019 in Mongolia. As of 2019, males and females had a sum of 40 434 and 24 572 DALYs, respectively, due to liver cancer. Of the total 65 006 DALYs due to liver cancer in 2019, 34% (22 386) and 31% (19 948) were attributed to hepatitis B and alcohol, respectively. YLLs outweighed YLDs accounting for more than 99% of DALYs with a male-to-female ratio of 1:6. In 2019, Mongolia had the highest age-standardized DALYs for all etiologies of liver cancer in the world. Alcohol-attributable DALYs were 28.9 times higher than the global average, and liver cancer due to hepatitis C and B were 21.5 and 10.9 times higher, respectively (**Supplementary Table 1**).

## Discussion

This study is the first comprehensive analysis of the estimated burden of liver cancer in Mongolia by its etiologies. Age-standardized incidence and death rates of all causes of liver cancer are higher in males than females during the study period, with a notable increase from 1990–2010, followed by a slight decline from 2010–2019. Liver cancer deaths accounted for about 10% of all causes of death in Mongolia in 2019. For males, death from liver cancer due to alcohol use and hepatitis B accounted for the majority of all liver cancer deaths. In contrast, deaths from liver cancer due to hepatitis C accounted for almost half of all liver cancer deaths for females. In terms of age distribution, more than half of all liver cancer deaths in males occurred between the ages of 50–69, while more than two-thirds of liver cancer deaths in females occurred in those over age 60.

According to the global liver cancer burden estimates, the highest age-standardized mortality rate was observed in Mongolia compared to the countries with the highest burden in the world (**Supplementary Figure 3**) (15). Alcohol consumption, hepatitis B, and C attributed liver cancer remained as one of the estimated top leading causes of death from 1990 to 2019 in Mongolia (4).

### Alcohol use

Among all causes, liver cancer due to alcohol use was the most frequent, especially for males, making Mongolia one of the highest alcohol-induced liver cancer observed in the world (16). Attribution of alcohol resulted in 786.6 age-standardized DALYs, 29 times higher than the global average of 26.1 in 2019. Alcohol consumption, particularly moderate and heavy drinking is associated with a higher risk of liver cancer (17). Its synergies with co-existing viral hepatitis infection contribute to liver cancer development (18). The higher percentage of alcohol-induced liver cancer is consistent with the overuse of alcohol in Mongolia. Armstrong et al. emphasized that the alcohol industry has been far more effective in promoting the “benefits” than the public health professionals have been in pointing out the “hazards” (21). Therefore, decreasing alcohol consumption could substantially contribute to a decline in the burden of liver cancer in Mongolia.

In 1992, Mongolia transitioned from a centrally planned and authoritarian system into a democratic nation which enabled a stronger market economy and foreign trade through the adoption of a new constitution (19, 20). Since then, the alcohol consumption per capita continuously increased up to 11 liters in 2015 and slightly decreased to 8.2 liters per capita in 2018 (21). As a result of foreign trade, investments, and privatization of businesses, alcohol production, and imports have increased along with its accessibility which potentially led to widespread alcoholism in the country (21). It is considered a social norm in Mongolia to conduct special occasions and activities with an alcohol-containing beverage. Heavy episodic drinking has a strong cultural drive during celebrations in urban areas while binge drinking is more likely to be explained as a coping mechanism of males to handle psychological distress, particularly unemployment or poverty in rural areas (22). Furthermore, family poverty contributes to gender disparity in children`s educational attainment. Lower completion and attendance rate in secondary schools with higher repetition and drop-out rates are reported among boys in Mongolia. Those boys are more likely to be engaged in hazardous child labor to generate income (23). The pattern of poverty and under-education has potential implications for unemployment and substance abuse which results in adverse health conditions.

### Viral hepatitis B and C

Viral hepatitis B and C are the main causes of liver cirrhosis and chronic liver inflammation that contribute to liver cancer development over a prolonged time (8, 24). These blood-borne and sexually transmitted infections are highly prevalent in Mongolia (8, 25–27). Over 95% of liver cancer cases are associated with hepatitis B or C infection (27). Contaminated syringes, medical devices, blood and blood products, and tools that break the skin are potential sources of infection (28, 29). Reusable glass syringe usage in both health facilities and home settings primarily contributed to the peak transmission of viral hepatitis infection up until the enforcement of disposable syringes in Mongolia in 1995 (30–32). Injection overuse for medication administration such as prescription medicines, antibiotics, vitamins, and antihypertensives was a common practice with an average of 13 injections per person in a year (33). A wider application of the injection treatment method up until today can be explained by the perceived effectiveness and preference for injectables over oral treatments among the population (33, 34). The majority of the affected population is over 50 years old and was born and lived before the availability of disposable syringes, as well as vaccination against hepatitis B in the country (30, 35).

Another source of the wider infection spread can be explained by blood transfusion, which was initiated in Mongolia in 1938 for the first time. The foundation of the National Centre for Blood Transfusion dates back to 1963 as a central station. However, donated blood and blood products have all gone under mandatory testing for transfusion-transmitted infection since the introduction in only 1997, except in emergency cases (6, 35, 36). The higher proportion of deferrals for transfusion-transmitted infection correlated with the higher incidence rate of hepatitis B and C, indicating the screening impact on the potential increase in diagnosis. Study results in the past suggested further improvements for screening tests which might have missed a larger proportion of viraemic donors (26, 37). It is a main risk factor among individuals with acute HCV in addition to iatrogenic infections due to medical exposures such as surgery and dental procedures.

Insufficient infection control practices and sterilization of equipment for medical, dental, and traditional medicine are particular concerns for the high prevalence of infection (6, 38–40). Although the infection control system is well-designated in the country, disinfection and sterilization of tools and medical equipment used at private dental clinics and obstetrics service providers are the main concerns (40–42). The registered risk factors for the transmission were identified as dental services followed by pregnancy and birth, injections, and surgeries (41). Cosmetics practices such as manicures, pedicures, body-piercing, and tattooing have a potentially higher risk for HCV infection acquisition (43–46). These high-risk factors for infection transmission and causes of high prevalence are suggested with further investigations needed to support the currently known evidence. Additionally, an identified population at intermediate and high risk of acquiring hepatitis C viral infection, namely patients with sexually transmitted diseases, female sex workers, men who have sex with men, mobile citizens, and people who inject drugs are to be further studied to address the current knowledge gap in the literature (47, 48).

Since the establishment of viral hepatitis surveillance in Mongolia in 1952, the highest burden was reported to occur around the 1970s (41, 49). In response, the national programme on the prevention and control of viral hepatitis has been implemented since 1988 to combat epidemics through the introduction of a vaccine against the hepatitis B virus, approval of the national strategy to combat viral hepatitis B and C, strengthening of infection control measures, screening of blood products, and standard operating procedures for testing, diagnosis, and treatment of viral hepatitis.

The National Immunization Programme introduced the hepatitis B vaccine into the routine immunization schedule in 1991 and was fully implemented in 1992. Nationwide coverage of birth doses is successfully administered through the strong immunization program which contributed to a significant decline in infection (41). Maintaining the current nationwide high vaccine coverage against the hepatitis B virus is the core indicator of the validation of viral hepatitis elimination in Mongolia (50). A recent study on the immunity against the hepatitis B virus among adolescents and young adults suggested a booster vaccine for adulthood, as the majority of the survey participants had a low anti-HBs titer which tends to decrease as age increases. Additional efforts on immunity surveillance and boosters may need to be considered to prevent breakthrough cases and to reduce asymptomatic carriers (51, 52). It is also an integral component of the elimination of mother-to-child transmission. The comprehensive tiered approach to elimination including neonatal Hepatitis B immunoglobulin (HBIG) administration within 12 hours of birth to infants born to HBV-positive mothers was introduced in 2019 (53). Further studies on the mother-to-child transmission rate would support the measurement of HBV seroprevalence, the efficacy of prevention measures, and the protection of high-risk infants (54). 287 HCV screening is available for pregnant women as part of the routine antenatal testing and the treatment guideline is specified in the procedure to eliminate the mother-to-child transmission of HIV, hepatitis B virus, and syphilis (55).

### Non-alcoholic steatohepatitis

NASH-induced liver cancer, primarily caused by metabolic risk factors is on the rise globally. Mongolia ranks second for NASH-related liver cancer attributable to high fasting plasma glucose in 2019. Lifestyle-related disorders such as diabetes - insulin resistance is mainly responsible for subclinical inflammation and liver damage that leads to liver cancer (56). One in three people aged 15–69 in Mongolia is at high risk for developing non-communicable diseases, with 49.4% of the population being obese and overweight. Of those, women are more likely to be overweight compared to men. The mean fasting plasma glucose level increased up to 5.9 mmol/L among the population in 2019 (57). Potential implications of the high fasting plasma glucose-induced NASH-related liver cancer should be explored in the country. Besides the high body mass index and HFPG level in women, wider usage of self-prescribed medications possibly accelerates liver damage, creating a more favorable environment for cancerous conditions (15).

Most liver cancer cases are diagnosed at a late stage. About 76.1% of the patients were diagnosed with stage III or IV cancer. More than half of these people died within one year of the diagnosis (58). Although subsidized testing and treatment options are available, a cascade of care services is insufficient with less than one-third of the hepatitis B and C positive cases going for confirmation testing and treatment enrolment. Only hepatitis C-confirmed patients have a higher treatment completion rate. In addition to the inadequate follow-ups, the financial burden on uninsured patients is a potential cause of late-stage diagnosis of liver cancer as the government subsidy is provided through public health insurance only (59). The lower survival rate after the diagnosis is consistent with the study findings. More than 99% of DALYs are attributed to YLLs due to mainly liver cancer caused by alcohol use (14 725) and hepatitis B (16 100) for men, and hepatitis C (9 590) for women.

### Initiatives and measures to combat liver cancer

In response to this epidemic, the Government of Mongolia combats liver cancer as a public health concern among the population through the implementation of the National program on the prevention and control of viral hepatitis 1988–2000, National program on prevention and control of communicable diseases 2002–2021, and National strategy of prevention and control for viral hepatitis 2010–2015 (60). Since 2017, the Healthy Liver Program 2017–2020, a national flagship program is being implemented, as well as an amendment to the national guidelines on HBV, HCV, and HDV screening, diagnosis, and treatment in 2018 and 2019 (61). The program aimed to strengthen the infection control and surveillance system, and provide evidence-based accessible, and quality care services to eliminate viral hepatitis as a primary cause of liver cancer and cirrhosis. It enabled highly effective treatment availability and accessibility at an affordable price for those covered by health insurance. However, testing and treatment of the uninsured population are to be ensured and the sustainability measures are to be further considered. As of 2020, 61.8 billion MNT (Mongolian Tugrik which is approximately 18 million USD) in funding was allocated to cover the costs of laboratory diagnosis, drugs, and liver transplantation services (59). The pronounced decline in death rates between 2010 and 2019 can be explained by these remarkable efforts. Continuation of the program is being implemented through the national action plan on “Healthy Liver Mongolia” 2022–2025 (62, 63). The main goal is to eliminate viral hepatitis by implementing early detection, diagnosis, and treatment, decentralization of care services, increasing the accessibility and quality of services, improving the surveillance and electronic registry system for chronic hepatitis, cirrhosis, and liver cancer, and reduce the infection transmission risks by 2025 (63). These measures are promising to increase the survival rate of liver cancer patients and decrease mortality, as well as its attributable causes.

This study is the first in-depth analysis of the liver cancer burden in Mongolia to explore different etiologies based on the GBD. However, due to the limitations of shared national data available to the global database, incomplete data may result in data accuracy concerns and potential bias. Other risk factors such as exposure to environmental pollution are not examined in this study which has a significant contribution to liver cancer in Mongolia according to a recent study (63). Moreover, the combined effects of these multiple etiologies are to be explored further in future studies.

## Conclusion

Mongolia achieved substantial milestones in the prevention and control of many communicable and non-communicable diseases from 1990 to 2019. However, liver cancer and alcohol use disorders significantly increased over the past years. The association between alcohol use and mortality, and quality of life among men is notable for its enduring impact on the burden of disease in Mongolia. Another main cause of liver cancer mortality, viral hepatitis remains a public health threat in the country. A comprehensive approach including accessible and affordable high-quality testing, treatment, follow-up, and chronic care services through a national flagship program with a strong foundation of the surveillance system is important in scaling up the care services capacity to reach the goal of elimination by 2030. Cross-cutting intervention measures integrate efforts at all levels to enable more effective and efficient responses to epidemics. In addition to secondary and tertiary prevention measures, primary prevention plays a crucial role. Routine and voluntary vaccination offers the most effective protection from hepatitis B infection for newborns, children, healthcare workers, and adults at risk or with chronic conditions. Although a well-established immunization program provides high vaccination coverage in the country, serologic testing and maintenance of post-vaccine immunity need to be ensured for better protection of the population. Health education is a necessary component of health promotion for liver cancer prevention which needs equivalent attention. Adoption of a healthy lifestyle such as enrollment in vaccination and screening programs, disengagement in high-risk behaviors including smoking, alcohol, drug abuse, medicine consumption, and unprotected sexual intercourse, maintenance of normal body mass index through regular exercise, balanced diet, and limited exposure to harmful chemicals in the environment builds up to the stronger foundation of public health measures while lowering individual`s risk factors for prolonged longevity. Based on the unique demographic and geographical characteristics, including various challenges ranging from individual access to medical services to availability of lifelong chronic care, as well as its adherence, tailored approaches for specific target populations are needed to tackle the liver cancer burden in Mongolia.

## Data Availability

The original contributions presented in the study are included in the article/**Supplementary Material**. Further inquiries can be directed to the corresponding author.
