# Supplementary material for: The burden of liver cancer in Mongolia from 1990–2019: a systematic analysis for the Global Burden of Disease Study 2019
Source: Front Oncol. 2024 Sep 3;14:1381173. doi: 10.3389/fonc.2024.1381173 (PMC11405307; doi:10.3389/fonc.2024.1381173)
Supplement: Supplementary file 1 [file DataSheet1.docx]

Appendix: supplementary results to “The burden of liver cancer in Mongolia, 1990-2019: a systematic analysis for the Global Burden of Disease Study 2019”

Table of Contents

[Supplementary table 1. Leading 20 countries in terms of age-standardized rate of DALYs in 2019 for both sexes. 3](#_Toc155349110)

[Supplementary table 2: Incidence of liver cancer due to hepatitis B in Mongolia for the years 1990-2019. 4](#_Toc155349111)

[Supplementary table 3: Prevalence of cirrhosis and other chronic liver diseases due to hepatitis B in Egypt and Mongolia, for the years 1990-2019. 5](#_Toc155349112)

[Supplementary table 4: Prevalence of cirrhosis and other chronic liver diseases due to hepatitis C in Egypt and Mongolia, for the years 1990-2019. 6](#_Toc155349113)

[Supplementary table 5: Incidence of liver cancer due to hepatitis B in Egypt and Mongolia, for the years 1990-2019. 7](#_Toc155349114)

[Supplemental table 6: Incidence of liver cancer due to hepatitis C in Egypt and Mongolia, for the years 1990-2019. 8](#_Toc155349115)

[Supplementary figure 1. Fraction of liver cancer death in Mongolia between 1990 and 2019 by sex 11](#_Toc155349116)

[Supplementary figure 2. Liver cancer mortality by its etiologies 12](#_Toc155349117)

[Supplementary figure 3. Comparison of 10 leading countries in terms of the age-adjusted mortality rate of liver cancer during 1990-2019 13](#_Toc155349118)

[Authors’ contributions 14](#_Toc155349119)

## Supplementary table 1. Leading 20 countries in terms of age-standardized rate of DALYs in 2019 for both sexes.

**
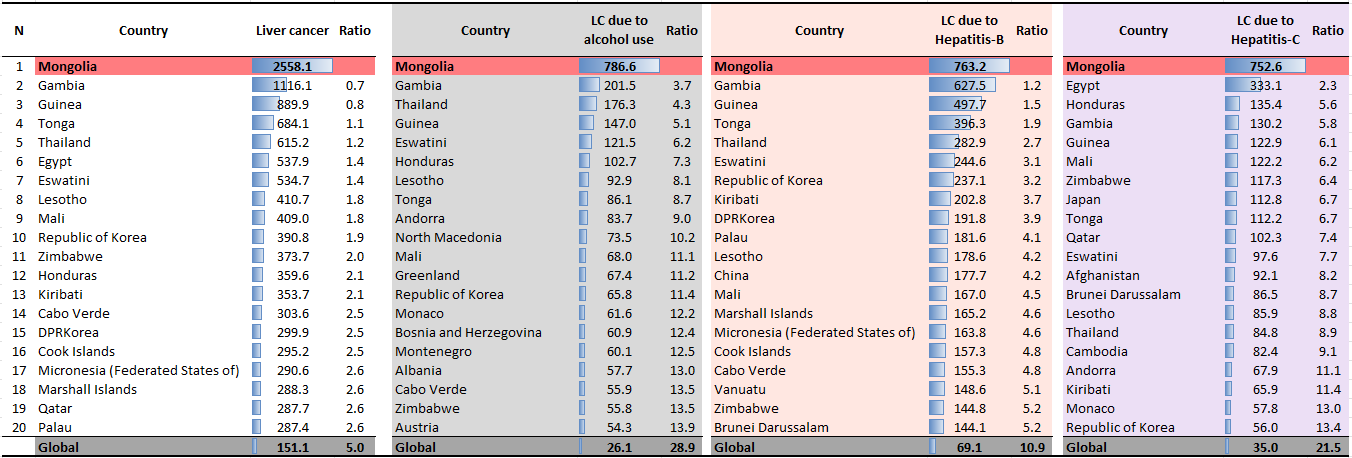
**

Ratio is between age-standardized DALY rate of Mongolia and other countries.

## Supplementary table 2: Incidence of liver cancer due to hepatitis B in Mongolia for the years 1990-2019.

| **measure** | **location** | **sex** | **age** | **cause** | **metric** | **year** | **value** |
| --- | --- | --- | --- | --- | --- | --- | --- |
| Incidence | Mongolia | Both | Age-standardized | Liver cancer due to hepatitis B | Rate | 1990 | 21.54741 |
| Incidence | Mongolia | Both | Age-standardized | Liver cancer due to hepatitis B | Rate | 1991 | 21.95404 |
| Incidence | Mongolia | Both | Age-standardized | Liver cancer due to hepatitis B | Rate | 1992 | 22.34859 |
| Incidence | Mongolia | Both | Age-standardized | Liver cancer due to hepatitis B | Rate | 1993 | 22.71608 |
| Incidence | Mongolia | Both | Age-standardized | Liver cancer due to hepatitis B | Rate | 1994 | 23.02252 |
| Incidence | Mongolia | Both | Age-standardized | Liver cancer due to hepatitis B | Rate | 1995 | 23.24789 |
| Incidence | Mongolia | Both | Age-standardized | Liver cancer due to hepatitis B | Rate | 1996 | 23.52451 |
| Incidence | Mongolia | Both | Age-standardized | Liver cancer due to hepatitis B | Rate | 1997 | 23.93442 |
| Incidence | Mongolia | Both | Age-standardized | Liver cancer due to hepatitis B | Rate | 1998 | 24.44136 |
| Incidence | Mongolia | Both | Age-standardized | Liver cancer due to hepatitis B | Rate | 1999 | 24.97839 |
| Incidence | Mongolia | Both | Age-standardized | Liver cancer due to hepatitis B | Rate | 2000 | 25.51862 |
| Incidence | Mongolia | Both | Age-standardized | Liver cancer due to hepatitis B | Rate | 2001 | 26.25545 |
| Incidence | Mongolia | Both | Age-standardized | Liver cancer due to hepatitis B | Rate | 2002 | 27.26075 |
| Incidence | Mongolia | Both | Age-standardized | Liver cancer due to hepatitis B | Rate | 2003 | 28.35879 |
| Incidence | Mongolia | Both | Age-standardized | Liver cancer due to hepatitis B | Rate | 2004 | 29.3444 |
| Incidence | Mongolia | Both | Age-standardized | Liver cancer due to hepatitis B | Rate | 2005 | 30.04193 |
| Incidence | Mongolia | Both | Age-standardized | Liver cancer due to hepatitis B | Rate | 2006 | 30.57761 |
| Incidence | Mongolia | Both | Age-standardized | Liver cancer due to hepatitis B | Rate | 2007 | 31.13754 |
| Incidence | Mongolia | Both | Age-standardized | Liver cancer due to hepatitis B | Rate | 2008 | 31.64536 |
| Incidence | Mongolia | Both | Age-standardized | Liver cancer due to hepatitis B | Rate | 2009 | 31.98587 |
| Incidence | Mongolia | Both | Age-standardized | Liver cancer due to hepatitis B | Rate | 2010 | 32.09694 |
| Incidence | Mongolia | Both | Age-standardized | Liver cancer due to hepatitis B | Rate | 2011 | 31.7873 |
| Incidence | Mongolia | Both | Age-standardized | Liver cancer due to hepatitis B | Rate | 2012 | 31.07916 |
| Incidence | Mongolia | Both | Age-standardized | Liver cancer due to hepatitis B | Rate | 2013 | 30.22207 |
| Incidence | Mongolia | Both | Age-standardized | Liver cancer due to hepatitis B | Rate | 2014 | 29.42001 |
| Incidence | Mongolia | Both | Age-standardized | Liver cancer due to hepatitis B | Rate | 2015 | 28.90501 |
| Incidence | Mongolia | Both | Age-standardized | Liver cancer due to hepatitis B | Rate | 2016 | 28.61588 |
| Incidence | Mongolia | Both | Age-standardized | Liver cancer due to hepatitis B | Rate | 2017 | 28.29381 |
| Incidence | Mongolia | Both | Age-standardized | Liver cancer due to hepatitis B | Rate | 2018 | 27.85907 |
| Incidence | Mongolia | Both | Age-standardized | Liver cancer due to hepatitis B | Rate | 2019 | 27.28154 |

## Supplementary table 3: Prevalence of cirrhosis and other chronic liver diseases due to hepatitis B in Egypt and Mongolia, for the years 1990-2019.

| **measure** | **location** | **sex** | **age** | **cause** | **metric** | **year** | **Egypt** | **Mongolia** | **Ratio** |
| --- | --- | --- | --- | --- | --- | --- | --- | --- | --- |
| Prevalence | Egypt | Both | Age-standardized | Cirrhosis and other chronic liver diseases due to hepatitis B | Rate | 1990 | 6471.209 | 7646.4832 | 1.18 |
| Prevalence | Egypt | Both | Age-standardized | Cirrhosis and other chronic liver diseases due to hepatitis B | Rate | 1991 | 6402.293 | 7640.0375 | 1.19 |
| Prevalence | Egypt | Both | Age-standardized | Cirrhosis and other chronic liver diseases due to hepatitis B | Rate | 1992 | 6336.708 | 7607.9686 | 1.20 |
| Prevalence | Egypt | Both | Age-standardized | Cirrhosis and other chronic liver diseases due to hepatitis B | Rate | 1993 | 6277.084 | 7558.2758 | 1.20 |
| Prevalence | Egypt | Both | Age-standardized | Cirrhosis and other chronic liver diseases due to hepatitis B | Rate | 1994 | 6226.116 | 7498.4893 | 1.20 |
| Prevalence | Egypt | Both | Age-standardized | Cirrhosis and other chronic liver diseases due to hepatitis B | Rate | 1995 | 6186.451 | 7436.8288 | 1.20 |
| Prevalence | Egypt | Both | Age-standardized | Cirrhosis and other chronic liver diseases due to hepatitis B | Rate | 1996 | 6135.122 | 7323.6542 | 1.19 |
| Prevalence | Egypt | Both | Age-standardized | Cirrhosis and other chronic liver diseases due to hepatitis B | Rate | 1997 | 6059.452 | 7134.5841 | 1.18 |
| Prevalence | Egypt | Both | Age-standardized | Cirrhosis and other chronic liver diseases due to hepatitis B | Rate | 1998 | 5977.325 | 6916.1705 | 1.16 |
| Prevalence | Egypt | Both | Age-standardized | Cirrhosis and other chronic liver diseases due to hepatitis B | Rate | 1999 | 5906.635 | 6714.1212 | 1.14 |
| Prevalence | Egypt | Both | Age-standardized | Cirrhosis and other chronic liver diseases due to hepatitis B | Rate | 2000 | 5865.248 | 6575.1438 | 1.12 |
| Prevalence | Egypt | Both | Age-standardized | Cirrhosis and other chronic liver diseases due to hepatitis B | Rate | 2001 | 5816.071 | 6464.8528 | 1.11 |
| Prevalence | Egypt | Both | Age-standardized | Cirrhosis and other chronic liver diseases due to hepatitis B | Rate | 2002 | 5727.663 | 6331.1984 | 1.11 |
| Prevalence | Egypt | Both | Age-standardized | Cirrhosis and other chronic liver diseases due to hepatitis B | Rate | 2003 | 5626.225 | 6193.9813 | 1.10 |
| Prevalence | Egypt | Both | Age-standardized | Cirrhosis and other chronic liver diseases due to hepatitis B | Rate | 2004 | 5537.817 | 6072.668 | 1.10 |
| Prevalence | Egypt | Both | Age-standardized | Cirrhosis and other chronic liver diseases due to hepatitis B | Rate | 2005 | 5488.374 | 5987.0089 | 1.09 |
| Prevalence | Egypt | Both | Age-standardized | Cirrhosis and other chronic liver diseases due to hepatitis B | Rate | 2006 | 5442.118 | 5906.5532 | 1.09 |
| Prevalence | Egypt | Both | Age-standardized | Cirrhosis and other chronic liver diseases due to hepatitis B | Rate | 2007 | 5361.207 | 5799.0281 | 1.08 |
| Prevalence | Egypt | Both | Age-standardized | Cirrhosis and other chronic liver diseases due to hepatitis B | Rate | 2008 | 5268.371 | 5682.695 | 1.08 |
| Prevalence | Egypt | Both | Age-standardized | Cirrhosis and other chronic liver diseases due to hepatitis B | Rate | 2009 | 5186.272 | 5574.761 | 1.07 |
| Prevalence | Egypt | Both | Age-standardized | Cirrhosis and other chronic liver diseases due to hepatitis B | Rate | 2010 | 5137.572 | 5493.3937 | 1.07 |
| Prevalence | Egypt | Both | Age-standardized | Cirrhosis and other chronic liver diseases due to hepatitis B | Rate | 2011 | 5076.742 | 5387.0218 | 1.06 |
| Prevalence | Egypt | Both | Age-standardized | Cirrhosis and other chronic liver diseases due to hepatitis B | Rate | 2012 | 4965.362 | 5218.6224 | 1.05 |
| Prevalence | Egypt | Both | Age-standardized | Cirrhosis and other chronic liver diseases due to hepatitis B | Rate | 2013 | 4836.757 | 5028.8159 | 1.04 |
| Prevalence | Egypt | Both | Age-standardized | Cirrhosis and other chronic liver diseases due to hepatitis B | Rate | 2014 | 4724.252 | 4857.1416 | 1.03 |
| Prevalence | Egypt | Both | Age-standardized | Cirrhosis and other chronic liver diseases due to hepatitis B | Rate | 2015 | 4661.14 | 4743.8927 | 1.02 |
| Prevalence | Egypt | Both | Age-standardized | Cirrhosis and other chronic liver diseases due to hepatitis B | Rate | 2016 | 4553.791 | 4687.5947 | 1.03 |
| Prevalence | Egypt | Both | Age-standardized | Cirrhosis and other chronic liver diseases due to hepatitis B | Rate | 2017 | 4419.515 | 4617.9347 | 1.04 |
| Prevalence | Egypt | Both | Age-standardized | Cirrhosis and other chronic liver diseases due to hepatitis B | Rate | 2018 | 4335.403 | 4490.6917 | 1.04 |
| Prevalence | Egypt | Both | Age-standardized | Cirrhosis and other chronic liver diseases due to hepatitis B | Rate | 2019 | 4263.194 | 4312.5534 | 1.01 |

## Supplementary table 4: Prevalence of cirrhosis and other chronic liver diseases due to hepatitis C in Egypt and Mongolia, for the years 1990-2019.

| **measure** | **location** | **sex** | **age** | **cause** | **metric** | **year** | **Egypt** | **Mongolia** | **Ratio** |
| --- | --- | --- | --- | --- | --- | --- | --- | --- | --- |
| Prevalence | Egypt | Both | Age-standardized | Cirrhosis and other chronic liver diseases due to hepatitis C | Rate | 1990 | 7156.178 | 9977.9967 | 1.394319 |
| Prevalence | Egypt | Both | Age-standardized | Cirrhosis and other chronic liver diseases due to hepatitis C | Rate | 1991 | 7004.744 | 9957.8716 | 1.42159 |
| Prevalence | Egypt | Both | Age-standardized | Cirrhosis and other chronic liver diseases due to hepatitis C | Rate | 1992 | 6864.294 | 9935.9298 | 1.44748 |
| Prevalence | Egypt | Both | Age-standardized | Cirrhosis and other chronic liver diseases due to hepatitis C | Rate | 1993 | 6740.632 | 9912.7403 | 1.470595 |
| Prevalence | Egypt | Both | Age-standardized | Cirrhosis and other chronic liver diseases due to hepatitis C | Rate | 1994 | 6639.755 | 9888.876 | 1.489344 |
| Prevalence | Egypt | Both | Age-standardized | Cirrhosis and other chronic liver diseases due to hepatitis C | Rate | 1995 | 6567.491 | 9864.7786 | 1.502062 |
| Prevalence | Egypt | Both | Age-standardized | Cirrhosis and other chronic liver diseases due to hepatitis C | Rate | 1996 | 6509.066 | 9833.0218 | 1.510666 |
| Prevalence | Egypt | Both | Age-standardized | Cirrhosis and other chronic liver diseases due to hepatitis C | Rate | 1997 | 6450.779 | 9791.5225 | 1.517882 |
| Prevalence | Egypt | Both | Age-standardized | Cirrhosis and other chronic liver diseases due to hepatitis C | Rate | 1998 | 6399.525 | 9748.8507 | 1.523371 |
| Prevalence | Egypt | Both | Age-standardized | Cirrhosis and other chronic liver diseases due to hepatitis C | Rate | 1999 | 6362.105 | 9713.6472 | 1.526798 |
| Prevalence | Egypt | Both | Age-standardized | Cirrhosis and other chronic liver diseases due to hepatitis C | Rate | 2000 | 6345.151 | 9694.3493 | 1.527836 |
| Prevalence | Egypt | Both | Age-standardized | Cirrhosis and other chronic liver diseases due to hepatitis C | Rate | 2001 | 6429.696 | 9697.4981 | 1.508236 |
| Prevalence | Egypt | Both | Age-standardized | Cirrhosis and other chronic liver diseases due to hepatitis C | Rate | 2002 | 6644.084 | 9723.1519 | 1.46343 |
| Prevalence | Egypt | Both | Age-standardized | Cirrhosis and other chronic liver diseases due to hepatitis C | Rate | 2003 | 6915.35 | 9770.0455 | 1.412806 |
| Prevalence | Egypt | Both | Age-standardized | Cirrhosis and other chronic liver diseases due to hepatitis C | Rate | 2004 | 7170.007 | 9836.9544 | 1.371959 |
| Prevalence | Egypt | Both | Age-standardized | Cirrhosis and other chronic liver diseases due to hepatitis C | Rate | 2005 | 7334.017 | 9922.6659 | 1.352965 |
| Prevalence | Egypt | Both | Age-standardized | Cirrhosis and other chronic liver diseases due to hepatitis C | Rate | 2006 | 7402.646 | 10115.134 | 1.366421 |
| Prevalence | Egypt | Both | Age-standardized | Cirrhosis and other chronic liver diseases due to hepatitis C | Rate | 2007 | 7426.871 | 10437.682 | 1.405394 |
| Prevalence | Egypt | Both | Age-standardized | Cirrhosis and other chronic liver diseases due to hepatitis C | Rate | 2008 | 7415.76 | 10791.468 | 1.455207 |
| Prevalence | Egypt | Both | Age-standardized | Cirrhosis and other chronic liver diseases due to hepatitis C | Rate | 2009 | 7378.119 | 11078.188 | 1.501492 |
| Prevalence | Egypt | Both | Age-standardized | Cirrhosis and other chronic liver diseases due to hepatitis C | Rate | 2010 | 7322.653 | 11198.978 | 1.529361 |
| Prevalence | Egypt | Both | Age-standardized | Cirrhosis and other chronic liver diseases due to hepatitis C | Rate | 2011 | 7162.946 | 11124.185 | 1.553018 |
| Prevalence | Egypt | Both | Age-standardized | Cirrhosis and other chronic liver diseases due to hepatitis C | Rate | 2012 | 6845.73 | 10928.84 | 1.596446 |
| Prevalence | Egypt | Both | Age-standardized | Cirrhosis and other chronic liver diseases due to hepatitis C | Rate | 2013 | 6429.444 | 10670.385 | 1.659612 |
| Prevalence | Egypt | Both | Age-standardized | Cirrhosis and other chronic liver diseases due to hepatitis C | Rate | 2014 | 5972.718 | 10407.097 | 1.742439 |
| Prevalence | Egypt | Both | Age-standardized | Cirrhosis and other chronic liver diseases due to hepatitis C | Rate | 2015 | 5534.434 | 10196.557 | 1.842385 |
| Prevalence | Egypt | Both | Age-standardized | Cirrhosis and other chronic liver diseases due to hepatitis C | Rate | 2016 | 5086.085 | 9977.9798 | 1.961819 |
| Prevalence | Egypt | Both | Age-standardized | Cirrhosis and other chronic liver diseases due to hepatitis C | Rate | 2017 | 4527.931 | 9787.2628 | 2.161531 |
| Prevalence | Egypt | Both | Age-standardized | Cirrhosis and other chronic liver diseases due to hepatitis C | Rate | 2018 | 3683.836 | 9681.9441 | 2.628223 |
| Prevalence | Egypt | Both | Age-standardized | Cirrhosis and other chronic liver diseases due to hepatitis C | Rate | 2019 | 2528.942 | 9600.7037 | 3.796332 |

## Supplementary table 5: Incidence of liver cancer due to hepatitis B in Egypt and Mongolia, for the years 1990-2019.

| **measure** | **location** | **sex** | **age** | **cause** | **metric** | **year** | **Egypt** | **Mongolia** | **Ratio** |
| --- | --- | --- | --- | --- | --- | --- | --- | --- | --- |
| Incidence | Egypt | Both | Age-standardized | Liver cancer due to hepatitis B | Rate | 1990 | 2.287667 | 21.54741 | 9.418945 |
| Incidence | Egypt | Both | Age-standardized | Liver cancer due to hepatitis B | Rate | 1991 | 2.278827 | 21.95404 | 9.633921 |
| Incidence | Egypt | Both | Age-standardized | Liver cancer due to hepatitis B | Rate | 1992 | 2.266612 | 22.34859 | 9.859908 |
| Incidence | Egypt | Both | Age-standardized | Liver cancer due to hepatitis B | Rate | 1993 | 2.251503 | 22.71608 | 10.08929 |
| Incidence | Egypt | Both | Age-standardized | Liver cancer due to hepatitis B | Rate | 1994 | 2.233903 | 23.02252 | 10.30597 |
| Incidence | Egypt | Both | Age-standardized | Liver cancer due to hepatitis B | Rate | 1995 | 2.214293 | 23.24789 | 10.49901 |
| Incidence | Egypt | Both | Age-standardized | Liver cancer due to hepatitis B | Rate | 1996 | 2.183031 | 23.52451 | 10.77608 |
| Incidence | Egypt | Both | Age-standardized | Liver cancer due to hepatitis B | Rate | 1997 | 2.137844 | 23.93442 | 11.19559 |
| Incidence | Egypt | Both | Age-standardized | Liver cancer due to hepatitis B | Rate | 1998 | 2.090432 | 24.44136 | 11.69202 |
| Incidence | Egypt | Both | Age-standardized | Liver cancer due to hepatitis B | Rate | 1999 | 2.052913 | 24.97839 | 12.1673 |
| Incidence | Egypt | Both | Age-standardized | Liver cancer due to hepatitis B | Rate | 2000 | 2.037357 | 25.51862 | 12.52535 |
| Incidence | Egypt | Both | Age-standardized | Liver cancer due to hepatitis B | Rate | 2001 | 2.056915 | 26.25545 | 12.76448 |
| Incidence | Egypt | Both | Age-standardized | Liver cancer due to hepatitis B | Rate | 2002 | 2.107247 | 27.26075 | 12.93666 |
| Incidence | Egypt | Both | Age-standardized | Liver cancer due to hepatitis B | Rate | 2003 | 2.175084 | 28.35879 | 13.03802 |
| Incidence | Egypt | Both | Age-standardized | Liver cancer due to hepatitis B | Rate | 2004 | 2.247465 | 29.3444 | 13.05666 |
| Incidence | Egypt | Both | Age-standardized | Liver cancer due to hepatitis B | Rate | 2005 | 2.31158 | 30.04193 | 12.99628 |
| Incidence | Egypt | Both | Age-standardized | Liver cancer due to hepatitis B | Rate | 2006 | 2.391618 | 30.57761 | 12.78532 |
| Incidence | Egypt | Both | Age-standardized | Liver cancer due to hepatitis B | Rate | 2007 | 2.502033 | 31.13754 | 12.4449 |
| Incidence | Egypt | Both | Age-standardized | Liver cancer due to hepatitis B | Rate | 2008 | 2.615575 | 31.64536 | 12.09881 |
| Incidence | Egypt | Both | Age-standardized | Liver cancer due to hepatitis B | Rate | 2009 | 2.705388 | 31.98587 | 11.82302 |
| Incidence | Egypt | Both | Age-standardized | Liver cancer due to hepatitis B | Rate | 2010 | 2.74442 | 32.09694 | 11.69535 |
| Incidence | Egypt | Both | Age-standardized | Liver cancer due to hepatitis B | Rate | 2011 | 2.729327 | 31.7873 | 11.64657 |
| Incidence | Egypt | Both | Age-standardized | Liver cancer due to hepatitis B | Rate | 2012 | 2.685471 | 31.07916 | 11.57308 |
| Incidence | Egypt | Both | Age-standardized | Liver cancer due to hepatitis B | Rate | 2013 | 2.630379 | 30.22207 | 11.48962 |
| Incidence | Egypt | Both | Age-standardized | Liver cancer due to hepatitis B | Rate | 2014 | 2.581168 | 29.42001 | 11.39795 |
| Incidence | Egypt | Both | Age-standardized | Liver cancer due to hepatitis B | Rate | 2015 | 2.55537 | 28.90501 | 11.31147 |
| Incidence | Egypt | Both | Age-standardized | Liver cancer due to hepatitis B | Rate | 2016 | 2.56695 | 28.61588 | 11.14781 |
| Incidence | Egypt | Both | Age-standardized | Liver cancer due to hepatitis B | Rate | 2017 | 2.576466 | 28.29381 | 10.98163 |
| Incidence | Egypt | Both | Age-standardized | Liver cancer due to hepatitis B | Rate | 2018 | 2.548217 | 27.85907 | 10.93277 |
| Incidence | Egypt | Both | Age-standardized | Liver cancer due to hepatitis B | Rate | 2019 | 2.494036 | 27.28154 | 10.93871 |

## Supplemental table 6: Incidence of liver cancer due to hepatitis C in Egypt and Mongolia, for the years 1990-2019.

| **measure** | **location** | **sex** | **age** | **cause** | **metric** | **year** | **Egypt** | **Mongolia** | **Ratio** |
| --- | --- | --- | --- | --- | --- | --- | --- | --- | --- |
| Incidence | Egypt | Both | Age-standardized | Liver cancer due to hepatitis C | Rate | 1990 | 11.29629 | 20.42318 | 1.807954 |
| Incidence | Egypt | Both | Age-standardized | Liver cancer due to hepatitis C | Rate | 1991 | 11.06387 | 20.37668 | 1.841731 |
| Incidence | Egypt | Both | Age-standardized | Liver cancer due to hepatitis C | Rate | 1992 | 10.85559 | 20.39588 | 1.878836 |
| Incidence | Egypt | Both | Age-standardized | Liver cancer due to hepatitis C | Rate | 1993 | 10.68047 | 20.45621 | 1.915291 |
| Incidence | Egypt | Both | Age-standardized | Liver cancer due to hepatitis C | Rate | 1994 | 10.54497 | 20.53992 | 1.94784 |
| Incidence | Egypt | Both | Age-standardized | Liver cancer due to hepatitis C | Rate | 1995 | 10.45718 | 20.62708 | 1.972528 |
| Incidence | Egypt | Both | Age-standardized | Liver cancer due to hepatitis C | Rate | 1996 | 10.398 | 20.92123 | 2.012043 |
| Incidence | Egypt | Both | Age-standardized | Liver cancer due to hepatitis C | Rate | 1997 | 10.34796 | 21.57861 | 2.0853 |
| Incidence | Egypt | Both | Age-standardized | Liver cancer due to hepatitis C | Rate | 1998 | 10.32018 | 22.50504 | 2.180682 |
| Incidence | Egypt | Both | Age-standardized | Liver cancer due to hepatitis C | Rate | 1999 | 10.32735 | 23.60072 | 2.285265 |
| Incidence | Egypt | Both | Age-standardized | Liver cancer due to hepatitis C | Rate | 2000 | 10.38155 | 24.78165 | 2.387085 |
| Incidence | Egypt | Both | Age-standardized | Liver cancer due to hepatitis C | Rate | 2001 | 10.63753 | 26.57868 | 2.498577 |
| Incidence | Egypt | Both | Age-standardized | Liver cancer due to hepatitis C | Rate | 2002 | 11.15542 | 29.2258 | 2.619874 |
| Incidence | Egypt | Both | Age-standardized | Liver cancer due to hepatitis C | Rate | 2003 | 11.80959 | 32.18756 | 2.725544 |
| Incidence | Egypt | Both | Age-standardized | Liver cancer due to hepatitis C | Rate | 2004 | 12.47427 | 34.91232 | 2.798748 |
| Incidence | Egypt | Both | Age-standardized | Liver cancer due to hepatitis C | Rate | 2005 | 13.02686 | 36.85687 | 2.829299 |
| Incidence | Egypt | Both | Age-standardized | Liver cancer due to hepatitis C | Rate | 2006 | 13.60544 | 38.24081 | 2.8107 |
| Incidence | Egypt | Both | Age-standardized | Liver cancer due to hepatitis C | Rate | 2007 | 14.32731 | 39.58205 | 2.762699 |
| Incidence | Egypt | Both | Age-standardized | Liver cancer due to hepatitis C | Rate | 2008 | 15.04007 | 40.71759 | 2.707273 |
| Incidence | Egypt | Both | Age-standardized | Liver cancer due to hepatitis C | Rate | 2009 | 15.59443 | 41.50916 | 2.661794 |
| Incidence | Egypt | Both | Age-standardized | Liver cancer due to hepatitis C | Rate | 2010 | 15.83951 | 41.8174 | 2.640069 |
| Incidence | Egypt | Both | Age-standardized | Liver cancer due to hepatitis C | Rate | 2011 | 15.80498 | 41.41176 | 2.620172 |
| Incidence | Egypt | Both | Age-standardized | Liver cancer due to hepatitis C | Rate | 2012 | 15.64913 | 40.41286 | 2.582435 |
| Incidence | Egypt | Both | Age-standardized | Liver cancer due to hepatitis C | Rate | 2013 | 15.41873 | 39.14764 | 2.538967 |
| Incidence | Egypt | Both | Age-standardized | Liver cancer due to hepatitis C | Rate | 2014 | 15.15735 | 37.97024 | 2.505072 |
| Incidence | Egypt | Both | Age-standardized | Liver cancer due to hepatitis C | Rate | 2015 | 14.91145 | 37.19977 | 2.494712 |
| Incidence | Egypt | Both | Age-standardized | Liver cancer due to hepatitis C | Rate | 2016 | 14.60094 | 36.61823 | 2.507936 |
| Incidence | Egypt | Both | Age-standardized | Liver cancer due to hepatitis C | Rate | 2017 | 14.25252 | 36.03835 | 2.528561 |
| Incidence | Egypt | Both | Age-standardized | Liver cancer due to hepatitis C | Rate | 2018 | 13.94487 | 35.53338 | 2.548132 |
| Incidence | Egypt | Both | Age-standardized | Liver cancer due to hepatitis C | Rate | 2019 | 13.64074 | 35.02042 | 2.56734 |


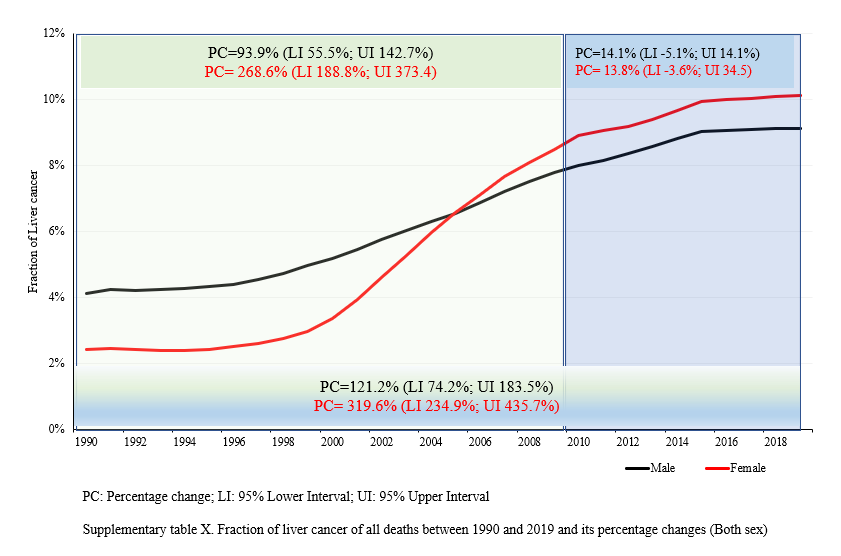


## Supplementary figure 1. Fraction of liver cancer death in Mongolia between 1990 and 2019 by sex


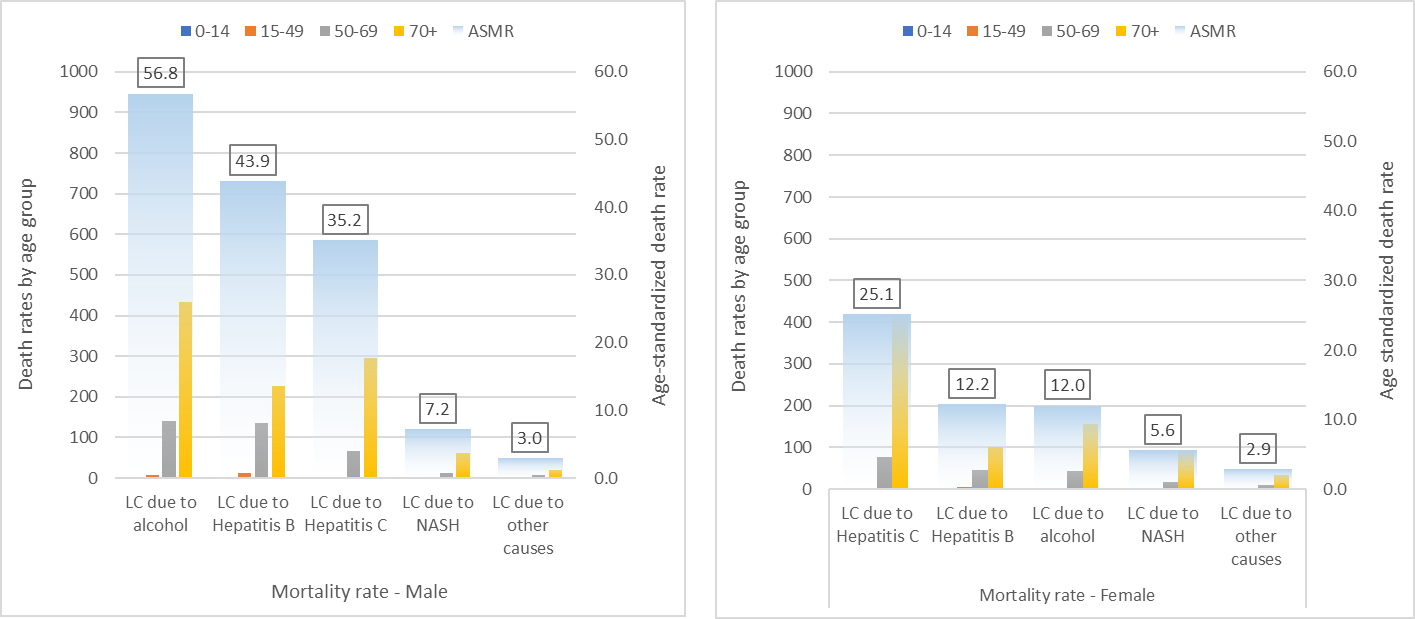


## Supplementary figure 2. Liver cancer mortality by its etiologies


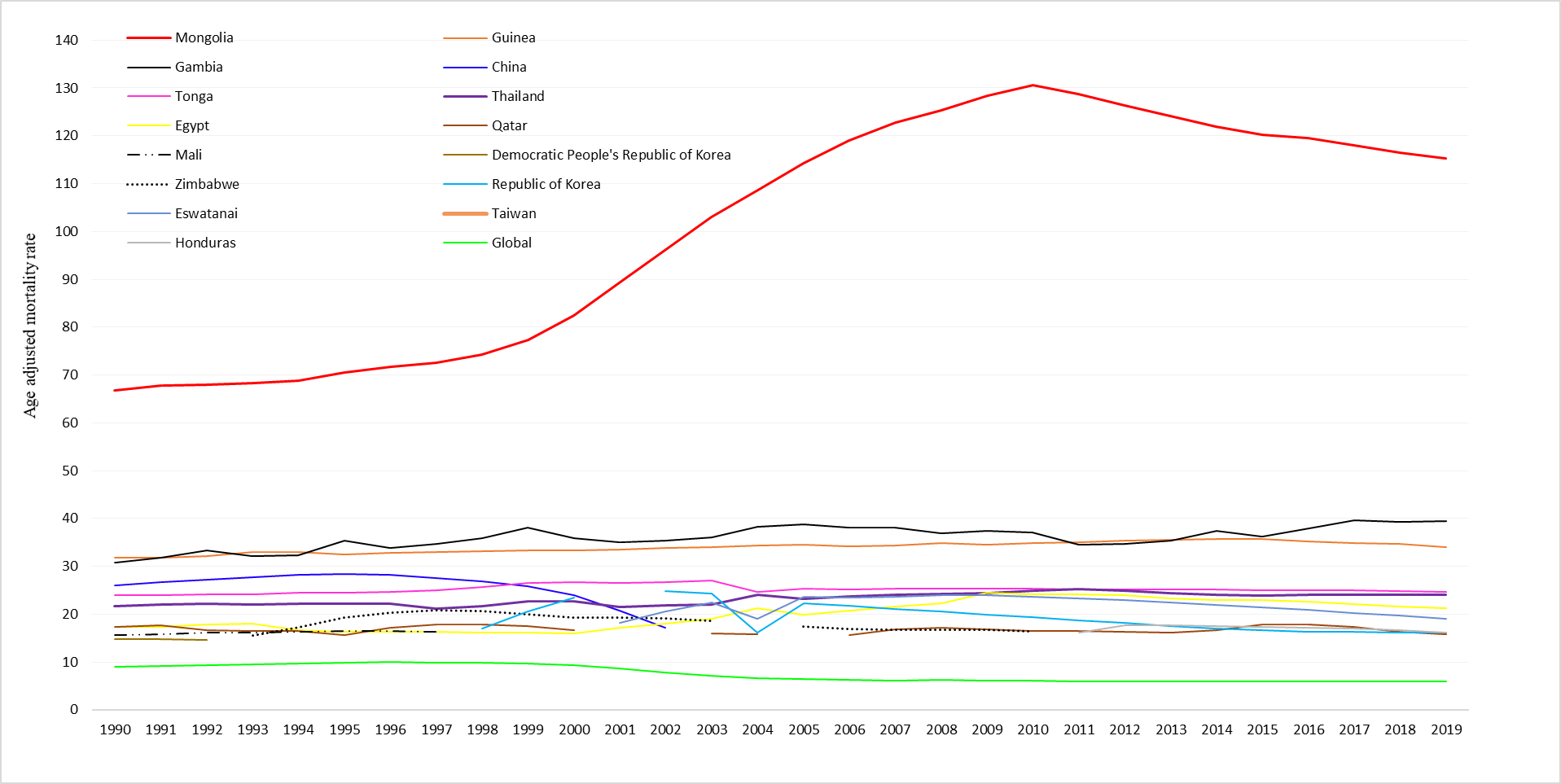


## Supplementary figure 3. Comparison of 10 leading countries in terms of the age-adjusted mortality rate of liver cancer during 1990-2019

## Authors’ contributions

**Providing data or critical feedback on data sources**

Oyundari Batsaikhan, Badral B D Davgasuren, Ali H Mokdad, Mohsen Naghavi, Anuzaya Purevdagva, and Amarzaya Sarankhuu

**Developing methods or computational machinery**

Oyundari Batsaikhan, Badral B D Davgasuren, Ali H Mokdad, Christopher J L Murray, and Mohsen Naghavi

**Providing critical feedback on methods or results**

Oyundari Batsaikhan, Odgerel Chimed-Ochir, Badral B D Davgasuren, Nicole Davis Weaver, Vanya Delgermaa, Chinburen Jigjidsuren, Tatsuhiko Kubo, Ali H Mokdad, Christopher J L Murray, Mohsen Naghavi, Erdenekhuu Nansalmaa, Anuzaya Purevdagva, and Uranchimeg Tsegmid

**Drafting the work or revising it critically for important intellectual content**

Oyundari Batsaikhan, Odgerel Chimed-Ochir, Badral B D Davgasuren, Nicole Davis Weaver, Vanya Delgermaa, Ryenchindorj Erkhembayar, Chinburen Jigjidsuren, Ali H Mokdad, Christopher J L Murray, Mohsen Naghavi, Erdenekhuu Nansalmaa, Oyuntsetseg Purev, Anuzaya Purevdagva, and Uranchimeg Tsegmid

**Managing the estimation or publications process**

Oyundari Batsaikhan, Badral B D Davgasuren, Nicole Davis Weaver, Ali H Mokdad, Christopher J L Murray, Mohsen Naghavi, and Amarzaya Sarankhuu
